# E-scooter accidents—epidemiology and injury patterns: 3-year results from a level 1 trauma center in Germany

**DOI:** 10.1007/s00402-024-05209-5

**Published:** 2024-02-17

**Authors:** Christoph Linhart, Ludwig Jägerhuber, Christian Ehrnthaller, Judith Schrempf, Adrian Cavalcanti Kußmaul, Carl Neuerburg, Wolfgang Böcker, Christopher Lampert

**Affiliations:** Department of Orthopaedics and Trauma Surgery, Musculoskeletal University Center Munich (MUM) University Hospital, LMU Munich, Marchioninistr. 15, 81377 Munich, Germany

**Keywords:** E-Scooter, Emergency department, Injuries, Brain trauma, Epidemiology, Alcohol

## Abstract

**Introduction:**

Since the introduction of e-scooters in Germany in 2019, they are becoming more and more popular and associated injuries have increased significantly. The aim of this study was to assess the injury patterns after e-scooter accidents.

**Materials and Methods:**

From May 2019 to October 2022, all consecutive patients who presented at our emergency department (ED) following e-scooter accidents were included in our study and retrospectively analyzed.

**Results:**

A total of 271 patients were included in our study. The mean age was 33 years. 38% of the patients were female and 62% were male. Most common injuries were traumatic brain injuries in 38% of the patients together with fractures affecting the upper limb (17%). An operative treatment was necessary in 40 patients. Most of the patients presented at night and about 30% were under the influence of alcohol.

**Conclusions:**

Our study shows one of the largest cohort of patients suffering e-scooter accidents in Europe. Compulsory helmet use, stricter alcohol controls and locking periods could contribute significantly to safety.

## Introduction

The trend toward urbanization is unbroken—also in Germany. However, metropolises are under strong pressure to transform themselves. There are many reasons for this: climate protection, digitalization and the desire for a better quality of life are at the forefront. However, metropolises have lost none of their appeal at the beginning of this century. On the contrary, while many regions are struggling with rural exodus, the big cities continue to grow.

This leads to a continuous increase in inner-city traffic volume and thus to space and environmental problems [12]. A change in transport policy is therefore indispensable. The city of the future needs an efficient local public transport system—as the basis of urban mobility. Sharing services complement bus and rail transport, by bicycle, scooter or even car. Increasingly, new drive concepts must be used within the framework of post-fossil mobility concepts. In Germany, e-scooters were approved in the major conurbations on 15 June 2019 as a component of this requirement in the course of the "Electric Mini-Vehicle Ordinance", for the use of which no helmet is compulsory and a minimum age of 14 years is required.

Studies from the USA, where e-scooters have been part of the urban landscape for years, point in particular to a high number of cases of fractures (26–32%) as well as head injuries (28–40%) [2, 18, 22]. These new injury patterns and accident mechanisms were confirmed from Europe and most recently also from Germany [14, 16]. For example, the work of Namiri et al. in the USA between 2014 and 2018 showed an increase in e-scooter related injuries of 222% and an increased hospitalization rate of 365% [18]. A retrospective study by Kobayashi et al. showed a mean injury severity score (ISS) of 5.9 in 103 patients over a 14-month period [11]. The influence of alcohol was investigated in the work of colleagues around Trivedi et al. and was reported to be 5% in 249 accidents [22], whereby the number of unreported cases is presumably significantly higher, as data from Germany have already shown [14].

Both the integration of e-scooters into German road traffic, which has so far only been partially scientifically investigated, and the ongoing debate about these alternative means of transport justify our study with the largest study population to date of 271 patients involved in accidents. In addition, our aim with this study is to analyze the frequency of emergency room consultations, new accident mechanisms and accident severity in e-scooter accidents at a level 1 trauma center in Germany and to derive recommendations for possible accident prevention for a safe use of these scooters.

## Materials and methods

All patients (18 years or older) who presented to the interdisciplinary emergency department in the city center of the university hospital LMU Munich in the period from May 1st 2019 to October 31st 2022 after accidents when riding e-scooters were included in this study. As there are 2 emergency departments of our hospital, we only focused on the one close to the city center. Patients were identified by checking the medical reports of the emergency department. Pedestrians that were injured as a result of an e-scooter accident were not included in this study. Demographic data (age, gender), date and time of presentation as well as type of presentation (by foot, by ambulance, accompanied by an emergency doctor or polytrauma via the resuscitation room), severity of the trauma using the Manchester Triage System, amount of blood alcohol (defined as ethanol content > 0,1 g/L), injury pattern and treatment type (surgical or conservative treatment, inpatient or outpatient) were retrospectively analyzed. The injury pattern was coded according to the body region and further differentiated according to the severity of the trauma and indication for surgical treatment.

## Results

From May 2019 until October 2022, data of 271 patients have been included in this retrospective study. The mean age of the analyzed population was 33 years (with a range from 18 to 64 years). 38% were female and 62% were male patients (see Table 1).

**Table 1:** Description of the study population

	Cases (*n*)	Percentage (%)
Total patients	271	
Gender		
Female	103	38
Male	168	62
Age		
Mean	33 (18–64 years)	
Time of accident		
4–8 a.m	25	9
8–12 a.m	39	14
12 a.m.–4 p.m	43	16
4–8 p.m	49	18
8 p.m.–12 a.m	51	19
12–4 a.m	64	24
Way of presentation		
By ambulance service	136	50
On their own	135	50
Alcohol consumption		
Yes	81	30
No	83	31
Unknown	107	39
Hospitalization	19	7

**Fig. 1:**
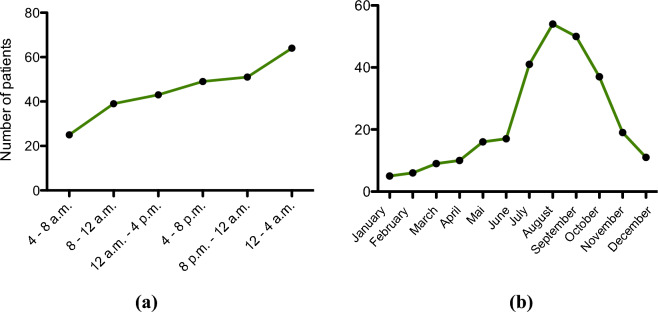
**a** Distribution of admissions based on time periods. **b** Distribution of admissions based on monthly frequency of e-scooter related accidents

With regard to the distribution of accidents by time of day, an increase in numbers during the day with the majority of patients presenting at the emergency department in the evening and at night could be observed. There was a continuous increase in patients suffering an e-scooter accident throughout the day with the majority of the patients presenting during the night time. A total of 51 patients (19%) presented to the emergency department from 8 p.m. to 12 a.m. and 64 patients (24%) from 12 to 4 a.m. Most of the accidents were documented during the summer months. A total of 145 accidents (54%) occurred from July till September (Fig. 1).

There were no significant differences in the way of presentation. A total of 135 patients (50%) presented to the emergency department on their own whereas 136 (50%) were referred by the ambulance service. A total of 8 patients (3%) were admitted to the hospital via the resuscitation room. According to the emergency system index (ESI), 116 patients (43%) were categorized as non urgent or minor injuries (ESI 4: *n* = 115, ESI 5: *n* = 1). Another 130 patients (48%) were categorized as potentially unstable (ESI 3) and 25 patients were categorized as urgent or received immediate care of an interdisciplinary team at the resuscitation room (ESI 2: *n* = 17, ESI 1: *n* = 8).

**Fig. 2:**
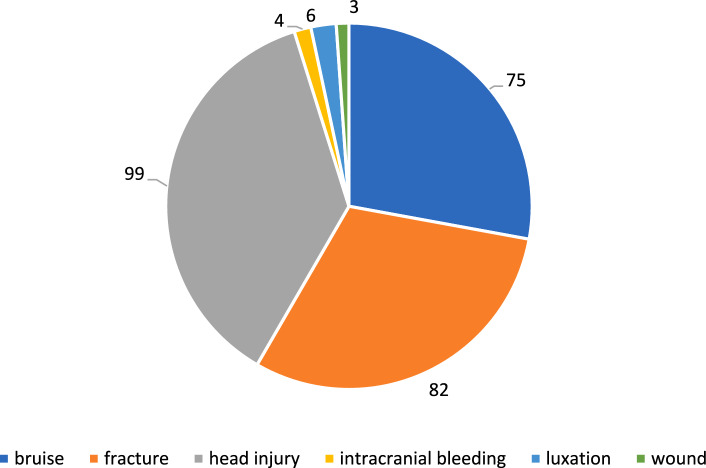
Injury patterns in e−scooter accidents

A total of 271 injuries were detected in our study population. Most of them affected the head with 104 patients (52%) suffering a traumatic brain injury. There were 100 traumatic brain injuries I° (37%) and an intracranial hemorrhage occurred in 4 patients (2%). In addition, 82 fractures (30%) and 6 joint luxations (2%) were diagnosed. Contusions were detected in 76 cases (28%). Of the 82 fractures, 47 involved the upper extremity (57%). Of these, 24 fractures affected hand or forearm (29%) and 23 fractures of the shoulder (28%) were recorded. The lower extremity was affected in 17 patients (21%) from which 6 fractures were located in the proximal femur or pelvis (7%) and 10 in the lower leg or foot (12%). Moreover, we could observe 12 maxillofacial fractures (orbital or nasal regions) (15%). Fractures of the spine were seen in 5 patients (6%). In our study population, each patient sustained 1.28 injuries on average (Fig. 2).

Further inpatient treatment immediately after presentation to the ED was necessary for 19 patients (7%), 12 of them needed immediate surgical treatment. Three other patients were admitted for neurological surveillance due to their intracranial hemorrhage and another patient for open head injury. On average, the patients stayed for 4 days (± 2,7 days) in hospital. Operative treatment was indicated in 40 cases (15%). Twenty-eight of them (70%) could be planned selectively, whereas 12 patients (30%) received surgery immediately after the treatment at the emergency department. A total of 42 fracture were treated conservatively (Table 2).

**Table 2:** Fracture patterns and frequency of surgical treatment

	Cases (*n*)	Percentage (%)	Surgical treatment n
Total	12	14.6%	
Head			
Midface	6	7.3%	1
Nasal bone	4	4.9%	–
Skull	2	2.4%	–
Total	5	6.1%	
Spine			
Cervical spine	2	2.4%	1
Lumbar spine	2	2.4%	–
Thoracic spine	1	1.2%	–
Total	47	57.3%	
Upper extremity			
Clavicula/AC joint	14	17.1%	9
Distal radius	8	9.8%	5
Proximal humerus	7	8.5%	4
Radial head	6	7.3%	–
Metacarpal/finger	5	6.1%	2
Carpal bone	4	4.9%	–
Scapula	2	2.4%	1
Olecranon	1	1.2%	1
Total	17	20.7%	
Lower extremity			
Ankle fractures	6	7.3%	5
Proximal femur	5	6.1%	5
Proximal tibia	2	2.4%	–
Metatarsal/toe	2	2.4%	–
Distal femur	1	1.2%	–
Chest			
Pelvis	1	1.2%	–
Rib	1	1.2%	–

In our study population, blood samples were taken in 164 patients (61%). A total of 81 accidents (30%) occurred under the influence of alcohol. In line with the increasing number of patients at night, 63 of them (78%) were registered in the emergency department between 10 p.m. and 6 a.m. The average amount of ethanol detected in the blood was 2.11 g/L (± 0.69 g/L). A closer look at the injury patterns of the intoxicated patients shows that 60 (74%) patients suffered a traumatic brain injury, with 3 (4%) patients having an intracranial hemorrhage. Furthermore, 19 (23%) fractures were diagnosed in intoxicated patients.

## Discussion

This study represents one of the largest study cohort of patients involved in e-scooter accidents in Europe. In total, we included 271 patients since Mai 2019, when e-scooter were first introduced in Germany.

Consistent with the previous literature about two thirds of the patients being affected in e-scooter accidents were male [7]. The most common injury pattern was a traumatic brain injury diagnosed in 38% of the patients. Kleinertz and Störmann et al. described in their studies an incidence of 54%, respectively, and 38% of head and facial injuries [10, 20]. Studies from USA, New Zealand, and Denmark also present similar numbers between 20 and 46% [4, 15, 21, 22]. The second largest study so far, which investigated a total of 249 patients in two trauma centers in Los Angeles showed nearly identical numbers with 40% head injuries and 2% of intracranial bleeding as well as fractures of the upper extremities being the second most common injury in 32% of the cases [22]. Our records do not disclose reliable information regarding the use of helmets. However, nearly all studies show a lack of helmet use with the absolute majority riding e-scooters without wearing a helmet [14, 21]. In Germany, as in most of the other countries, there is no obligation to wear a helmet. Harbrecht and Heuer could show in their studies that only 0–2% of the riders wear helmets [8, 9]. Seeing that high number of brain and face injuries, it needs to be discussed to provide helmets when renting e-scooters. In a study from Australia, 46% of the injured e-scooter riders wore a helmet and they could show a significant reduction of severe brain injuries [17].

Nearly every third patient suffered a fracture with more than half of them affecting the upper limb. Uluk et al. described in their study in Berlin similar numbers with fractures of the upper limb most frequently affected [23]. In contrast to Uluk et al., the number of fractures of the lower extremities was low. However, this could also be shown in the other studies. There are also a few studies describing different numbers with close percentages and even higher numbers of involvement of the lower limbs [5, 7]. The combination of head and neck injuries with injuries of the upper extremity suggests that patients were trying to catch themselves before landing [6]. When comparing the data from our study with previous studies investigating injury patterns after e-cooter accidents, there is a wide range in terms of need for surgical care and hospitalization rate [3, 13, 17, 21, 22]. Compared to our results, Bascone et al. describe in their study of 167 patients significant higher rates with a total of 117 fractures, (70%) of which 85 patients (50%) required surgical treatment and 105 patients (63%) were hospitalized [3]. It is also worth mentioning that more people suffer injuries due to the use of e-scooters as assumed in our study, because collateral damages like pedestrians were not included.

The influence of alcohol appears to play a major role in traumatic injuries after e-scooter accidents. Although in Germany the blood alcohol limit for e-scooter riders is below 0,47 g/L, about 30% of the accidents happened under the influence of alcohol with an average of 2,11 g/L. Current literature describe similar numbers with high rates of intoxicated riders [10, 14]. However, compared to the UK, only a minority of the reported cases were associated with the influence of alcohol (7.4%), although riders were also predominantly male and aged between 18 and 35 [1]. We could observe a change in the injury pattern of intoxicated riders, suffering a traumatic brain injury twice as often. This is also observed in other studies, hypothesizing that the consumption of alcohol impairs the capability to fall on the outstretched arm to protect from falling on the head [19]. Fittingly, the majority of the patients presented at night in the emergency department [20]. Also the proportion of young patients aged 18–40 was very high. However, in contrast to the previously described literature, the largest number of patients presented to the emergency department after midnight till 4 o’clock in the morning. This could be due to the central location in the city center near the nightlife district of our hospital. Obviously, the majority of accidents happen when the patients want to drive home under the influence of alcohol. In order to prevent this, locking periods should be discussed.

In a recently published study, comparing e-scooter accidents with e-bike and bicycle accidents from Germany, significant differences in injury patterns and driving under the influence of alcohol could be observed. In addition, the insufficient wearing of a helmet with a significant increase in head injuries in e-scooter accidents is once more to be emphasized [16]. Compared to bicycles, the lack of experience and the use mainly for leisure seem to contribute to the high injury and hospitalization rates [7].

This study also has some limitations related to its retrospective nature. First, relevant information, such as the wearing of a helmet, was not consistently recorded to provide a reliable conclusion. Similarly, information on the trauma mechanism (self-inflicted, collision with pedestrians or other vehicles) is lacking. Second, because of the inclusion of patients based on the medical history collected, it cannot be ruled out that data collection is incomplete. In addition, alcohol levels were only measured in about 60% of the patients taking into account the time of the accident, trauma mechanism and clinical hint of alcohol consumption, so that the relative number of accidents under the influence of alcohol can be assumed to be lower. However, a large sample was provided, and the data obtained represent comparable and nearly identical rates and outcomes to those reported in the previously described literature. Nevertheless, the number of patients could be significantly higher, but the evaluation was carried out during the Corona pandemic and, in addition, e-scooters were temporarily blocked during the Covid pandemic in Munich.

## Conclusion

Our study represents one of the largest patient cohorts involved in e-scooter accidents in Europe and provides valuable insights into injury patterns of this emerging mode of transportation. The findings emphasize the prevalence of traumatic brain injuries and fractures of the upper extremities as well as the significant role of alcohol. Moreover, the study underscores the higher incidence of accidents during nighttime often tied to alcohol consumption. While recognizing study limitations, our findings emphasize the importance of safety measures in the use of e-scooters. These include public health campaigns promoting helmet usage and potential policy interventions aimed at curbing alcohol-related accidents during late-night hours. It should be discussed to introduce blocking periods so that e-scooter renting is not possible during high-risk hours such as from 10 p.m. to 4 a.m. These steps are essential as e-scooter adoption continues to grow worldwide, prioritizing the safety of riders and pedestrians.
